# Natural killer cell immune synapse formation and cytotoxicity are controlled by tension of the target interface

**DOI:** 10.1242/jcs.258570

**Published:** 2021-04-15

**Authors:** Daniel Friedman, Poppy Simmonds, Alexander Hale, Leoma Bere, Nigel W. Hodson, Michael R. H. White, Daniel M. Davis

**Affiliations:** 1 The Lydia Becker Institute, Faculty of Biology, Medicine and Health, University of Manchester, Core Technology Facility building, 46 Grafton Street, Manchester M13 9NT, United Kingdom; 2 BioAFM Facility, Faculty of Biology, Medicine and Health, Stopford Building, University of Manchester, Oxford Road, Manchester M13 9PT, United Kingdom; 3 Michael Smith Building, Faculty of Life Sciences, University of Manchester, Manchester M13 9PT, United Kingdom

**Keywords:** Cellular activation, Cytotoxicity, Disease, Immune synapse, Mechanosensitivity, Natural killer cell

## Abstract

Natural killer (NK) cells can kill infected or transformed cells via a lytic immune synapse. Diseased cells may exhibit altered mechanical properties but how this impacts NK cell responsiveness is unknown. We report that human NK cells were stimulated more effectively to secrete granzymes A and B, FasL (also known as FasLG), granulysin and IFNγ, by stiff (142 kPa) compared to soft (1 kPa) planar substrates. To create surrogate spherical targets of defined stiffness, sodium alginate was used to synthesise soft (9 kPa), medium (34 kPa) or stiff (254 kPa) cell-sized beads, coated with antibodies against activating receptor NKp30 (also known as NCR3) and the integrin LFA-1 (also known as ITGAL). Against stiff beads, NK cells showed increased degranulation. Polarisation of the microtubule-organising centre and lytic granules were impaired against soft targets, which instead resulted in the formation of unstable kinapses. Thus, by varying target stiffness to characterise the mechanosensitivity of immune synapses, we identify soft targets as a blind spot in NK cell recognition.

This article has an associated First Person interview with the co-first authors of the paper.

## Abstract (precis)

**Highlighted Article:** Analyses of the impact of altered target rigidity reveal that soft targets expressing activating ligands do not induce polarisation of natural killer cellular machinery required for cytolysis.

## INTRODUCTION

Natural killer (NK) cells use a variety of germline-encoded activating and inhibitory receptors to detect signs of disease in other cells. If the balance of signalling from receptors favours cellular activation, an activating immune synapse (IS) assembles at the cell contact (Davis et al., 1999; Orange, 2008). The IS involves dynamic spatial organisation of surface receptors, signalling proteins and organisation of intracellular cytoskeletal elements (Bromley et al., 2001; Brown et al., 2012; Comrie et al., 2015). In the mature IS, polarisation of the microtubule-organising centre (MTOC), together with perforin-rich granules, towards the cell-cell interface is a critical requirement for directed cytotoxic granule expulsion. Remodelling of filamentous (F)-actin at the synapse results in an accumulation at the periphery and an opening up of the meshwork at the centre (Brown et al., 2011; Rak et al., 2011). This rearrangement forms channels in the F-actin network through which granules translocate before fusing with the synaptic membrane (Mace and Orange, 2014). The assembly of a lytic synapse is carefully regulated to ensure cytotoxicity is not directed against healthy tissue.

Mechanotransductive signalling pathways play an important role in a variety of cellular processes from embryonic development (Keller et al., 2003) to wound healing (Cox and Erler, 2011; Kuijpers et al., 1997). Several lines of evidence indicate the importance of mechanosensitive pathways in T-cell recognition. Mechanosensitive signalling impacts, for example, the discrimination between high and low potency peptides in CD8^+^ T cell recognition (Das et al., 2015; Liu et al., 2016). The importance of myosin contractility in CD8^+^ T-cell function was demonstrated by shRNA-mediated knockdown of the myosin II heavy chain, which resulted in reduced killing of B16 target cells (Basu et al., 2016). In addition, traction force microscopy measurements in the Jurkat T-cell line have established that actin polymerisation and myosin contractility contribute to the development of force at the cell interface (Hui et al., 2015). Granule secretion from CD8^+^ T cells has been shown to rely on the local application of force adjacent to membrane adhesion sites (Andzelm et al., 2007; Basu et al., 2016). However, these data have relied on chemical manipulations of cells or used planar substrates with varying stiffness; there has not been a direct test of how immune cell responses vary against three-dimensional spherical stimulants with defined stiffness.

During their lifetime, NK cells infiltrate a variety of tissues with differing mechanical properties, exposing them to large variations in matrix stiffness in the process. Atomic force microscopy (AFM) experiments have revealed that lung tissue is in the range of 2 kPa (Wells, 2013), muscle 10 kPa and cartilage 30 kPa, whereas cells developing in the bone marrow are exposed to substrate rigidities in the range of ∼25-100 kPa (Jansen et al., 2015; Discher et al., 2009). Inflamed and diseased tissues also present with chronically altered tissue rigidities (Suresh, 2007; Previtera and Sengupta, 2015). These changes depend on several factors, including dysregulated vasculature and variation in the extracellular matrix composition (Padera et al., 2004; Muiznieks and Keeley, 2013). Fibrosis is a key component of multiple diseases affecting millions of people, including liver cirrhosis, pulmonary fibrosis and arthritis (Friedman et al., 2013). Importantly, AFM measurements on fibrotic tissue reveal a characteristic tissue stiffening with elastic modulus values of 17 kPa, compared to 2 kPa for normal tissue (Lupher and Gallatin, 2006; Wells, 2013). Liver scarring and fibrosis are strongly coupled with hepatocyte death, and NK cell-mediated killing of hepatocytes has been reported (Ochi et al., 2004; Ishiyama et al., 2006).

Alongside changes to the rigidity of the surrounding matrix, cells themselves exhibit a variety of stiffnesses, ranging from 0.2 kPa to 10 kPa (Cross et al., 2007; Suresh, 2007). The mechanical properties of a cell change during oncogenesis, with primary solid tumour cells typically stiffer than the healthy cells from which they arise (Paszek et al., 2005). However, a significant decrease in stiffness is correlated with metastatic potential, with one study having found a 73% reduction in stiffness following metastasis (Cross et al., 2007; Guck et al., 2005; Hou et al., 2009). Viral infection also induces cortical actin restructuring, with one strain of rubella virus accounting for an increase of 17.8% in Young's modulus, a measure of substrate stiffness (Kräter et al., 2018). Thus, stiffening or softening of cells within tissues may be indicative of transformation. Whether there is a direct link between changes in tissue and/or cellular stiffness and NK cell responsiveness is currently untested.

Here, we set out to examine the impact of altered target rigidity on NK cell function. Using polyacrylamide substrates coated with monoclonal antibodies (mAb) against NKp30 (also known as NCR3) and lymphocyte function-associated antigen-1 (LFA-1, also known as ITGAL) to mimic the activating target interface, we demonstrate that NK cell degranulation and secretions of IFNγ, granzyme A and B, granulysin and Fas ligand (FasL, also known as FasLG) were enhanced on stiffer substrates. Using gel microbeads of varying stiffness as surrogate targets, we observed that interactions between NK cells and soft targets were typically asymmetrical and unstable. In contrast, stiff beads caused MTOC and lytic granule polarisation accompanied by strong degranulation. Thus, we demonstrate that target stiffness plays an important role in the formation of the NK cell synapse and downstream cellular activation.

## RESULTS

### Synthesis and characterisation of hydrogel substrates

**Fig. 1.**
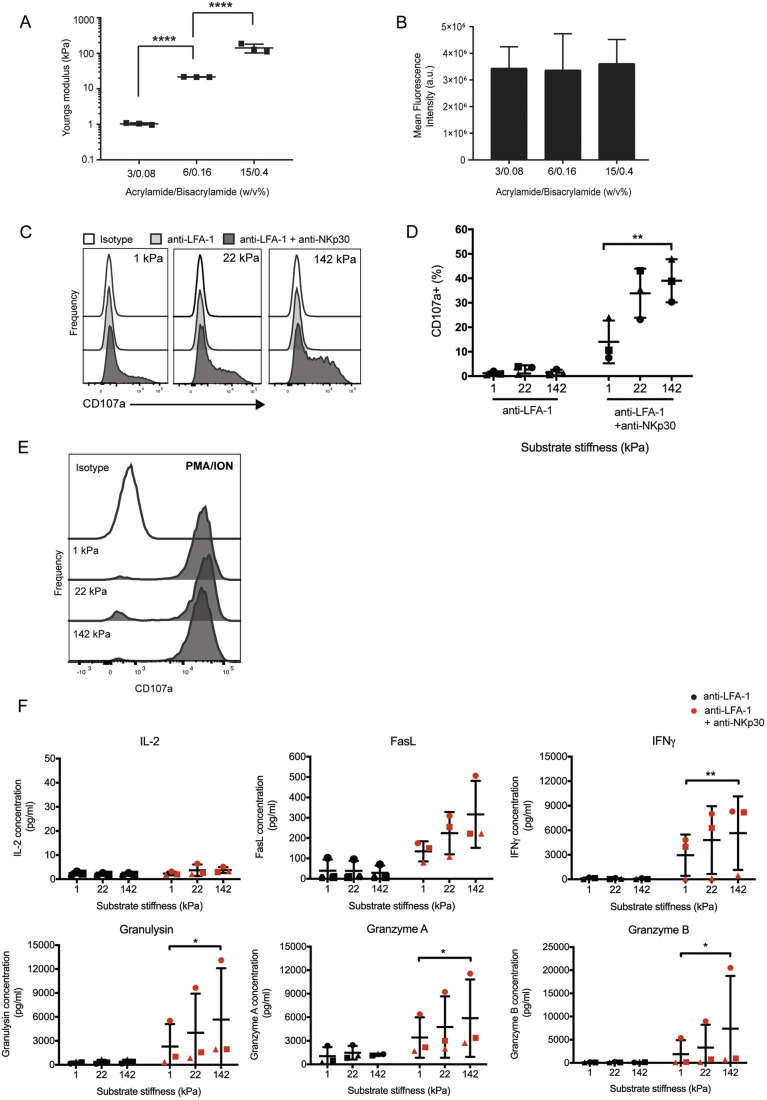
**NK cell cytotoxicity is sensitive to changes in substrate stiffness.** (A) Measurements of the Young's modulus (mechanical stiffness) of polyacrylamide gels synthesised from varying ratios of acrylamide and bis-acrylamide. Ten force curves were acquired from five different areas on the gel surface, and the elastic modulus values were averaged. Each data point represents an individual experiment independently analysed (*n*=3). (B) Quantification of anti-NKp30 mAb at the gel surface. Bound anti-NKp30 mAb at the gel surface was detected using an anti-IgG1-AF488 mAb. Three different areas of the gel surface were imaged and fluorescence intensities were quantified and averaged (*n*=5). (C) Primary NK cells were seeded on gels coated with anti-LFA-1 mAb alone or in combination with anti-NKp30 mAb, and incubated at 37°C for 4 h. Cells were stained for surface expression of CD107a and analysed by flow cytometry. Histograms shown are from one donor representative of three independent donors. (D) Quantification of CD107a^+^ cells. Data points of different shapes represent different donors (*n*=3). (E) Primary NK cells were seeded on anti-LFA-1 mAb coated hydrogels for 3 h at 37°C, treated with both PMA and ionomycin (ION), and left for a further 1 h. Cells were stained for surface expression of CD107a and analysed by flow cytometry. Histograms are from one representative donor out of three. (F) Primary NK cells were seeded on gels coated with anti-LFA-1 mAb alone or in combination with anti-NKp30 mAb, and left at 37°C for 24 h. Protein secretions in the supernatant were quantified using a cytokine bead array for (from left to right; first and second row) IL-2, Fas ligand (FasL), IFNγ, granulysin, granzyme A and granzyme B by analysis with flow cytometry. Cytokine concentrations were extrapolated from a standard curve for each. Data points of different shapes represent different donors (*n*=3). Data are mean±s.d. *P<0.05, **P<0.01, ****P<0.0001 (one-way ANOVA with Tukey's multiple comparisons). a.u., arbitrary units.

NK cells play a crucial role in eliminating malignant and diseased cells, but whether or not the rigidity of target cells affects NK cell cytotoxicity has not been widely studied. To test this, polyacrylamide substrates were synthesised to mimic the activating target interface covering a range of rigidities. By altering the final concentration of acrylamide/bis-acrylamide, substrates were produced with three different Young's moduli of 1±0.1, 22±1 and 142±35 kPa, as assessed by AFM (Fig. 1A). These values represent a range of physiologically relevant rigidities spanning soft, medium and stiff tissues, respectively (Xu et al., 2012; Nikolaev et al., 2014; Buxboim et al., 2010). To activate NK cells and induce assembly of a cytotoxic synapse, hydrogel surfaces were coated with monoclonal IgG1κ antibodies equilibrated across the different surfaces (Fig. 1B).

### NK cell degranulation efficiency is modified by the rigidity of the target substrate

To test whether substrate stiffness influences NK cell degranulation, NK cells were placed on 1, 22 and 142 kPa substrates coated with anti-LFA-1 mAb alone or with anti-LFA-1 plus anti-NKp30 mAb. After 4 h, the percentage of NK cells degranulating [as determined by CD107a (also known as LAMP1) expression on the cell surface] improved with increasing substrate stiffness (14±9%, 34±10% and 39±9%, on 1, 22 and 142 kPa surfaces, respectively; Fig. 1C,D; Fig. S1A). NK cells on the softer substrates were not impaired in their ability to respond, as, in the presence of phorbol 12-myristate 13-acetate (PMA) and ionomycin (soluble stimulation), all cells degranulated to the same extent, irrespective of the underlying substrate stiffness (Fig. 1E; Fig. S1B). Thus, increasing substrate stiffness results in enhanced degranulation, highlighting the tension of the target interface as an important parameter in facilitating efficient lytic granule secretion by NK cells.

### NK cell cytokine secretions are enhanced on stiffer substrates

Next, we set out to determine whether NK cell cytokine secretions were modulated by the stiffness of the activating substrate. To test this, primary NK cells were placed on 1, 22 and 142 kPa surfaces coated with anti-LFA-1 or anti-LFA-1 plus anti-NKp30 mAb. After 24 h, supernatants were collected and a range of pro-inflammatory secretions were quantified using a cytokine bead array. Little, if any, IL-2 was secreted in response to ligation of LFA-1 and NKp30. However, IFNγ, FasL, granzyme A and B and granulysin were all secreted. Importantly, secretions increased with substrate stiffness, with high levels of IFNγ, FasL, granzyme A and B, and granulysin all being secreted when NK cells were interacting with the stiffest 142 kPa substrates (Fig. 1F). By plotting the fold change in the IFNγ secretion of each donor, there was a 1.6 and 4.5-fold change in secretion for 22 kPa and 142 kPa surfaces, respectively, in comparison to 1 kPa surfaces (Fig. S1C).

### Synthesis and characterisation of gel microbeads

Degranulation at the IS occurs following successful polarisation of the MTOC and lytic granules towards the NK cell-target interface. Here, granules fuse with the synaptic membrane, and their contents are expelled into the synaptic cleft as supramolecular attack particles (Ambrose et al., 2020; Bálint et al., 2020). It is currently unclear how altering substrate stiffness impacts these different stages of NK cell activation, which lead up to secretion. Although flat gel substrates are useful for studying effector functions, examination of organelle and protein polarisation to the synapse is challenging. Capturing images in a defined *z*-plane in the very initial moments of cell interaction with the two-dimensional substrate excludes intracellular structures beyond a specific distance above the focal plane. We therefore sought to synthesise gel microbeads as a more physiological representation of a target cell than a flat two-dimensional surface, in which the stiffness could be altered in a controlled manner and the three-dimensional aspects of NK cell activation accurately assessed.

**Fig. 2.**
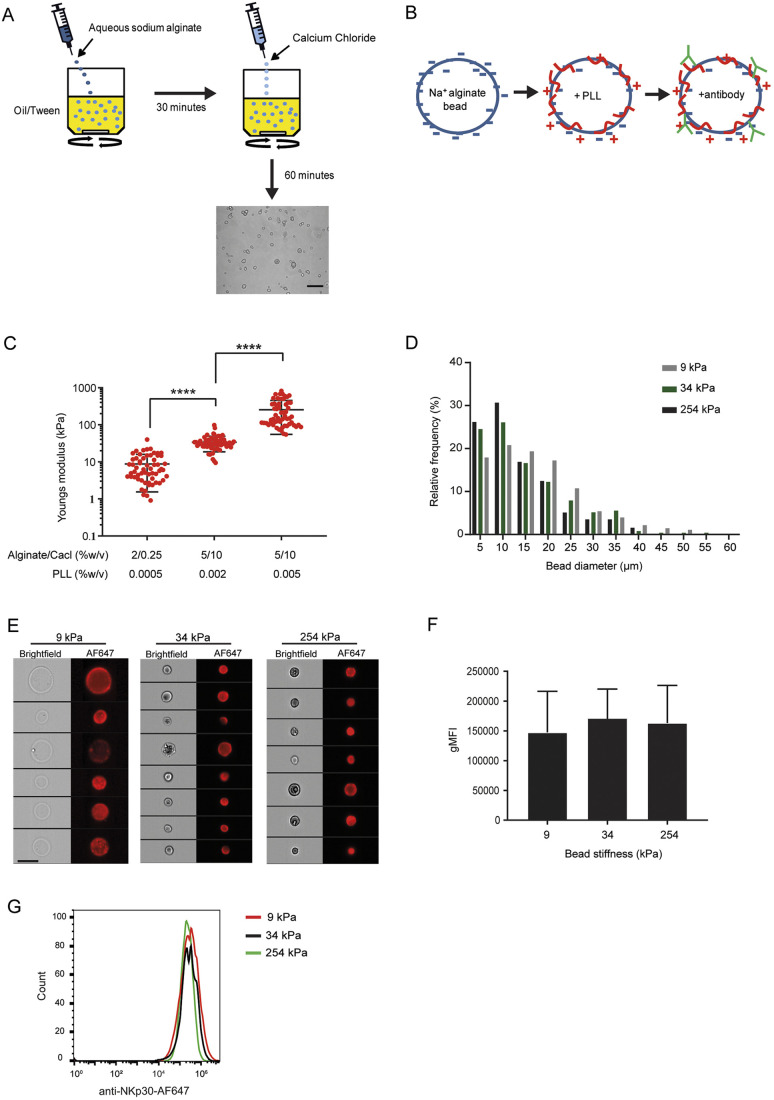
**Synthesis of sodium alginate gel beads for use as surrogate cell targets.** (A) Schematic demonstrating alginate bead synthesis, including a representative bright-field image. (B) Schematic showing electrostatic charges on the bead surface following incubation with PLL and mAb. (C) By altering the PLL, alginate and calcium chloride concentrations, three distinct bead rigidities were obtained. Each data point represents the mean stiffness (obtained from ten force curves) from an individual bead acquired by AFM. Stiffness profiles of beads (15-20 per experiment) were obtained and results from three independently analysed experiments are shown. (D) The diameters of 50 beads from each stiffness was measured and the relative frequency plotted. (E) Representative bright-field and fluorescent images from three independent experiments of 9 kPa (soft), 34 kPa (medium) and 254 kPa (stiff) beads coated with 50, 10 and 10 μg/ml anti-NKp30-AF647 mAb, respectively, acquired using imaging flow cytometry. (F) Equilibrated geometric mean fluorescence intensity (gMFI) values of soft, medium and stiff beads coated with anti-NKp30-AF647 (*n*=3). (G) Representative histogram plots from E. Data are mean±s.d. ****P<0.0001 (one-way ANOVA with Tukey's multiple comparison test). Scale bars: 50 μm (A); 10 μm (E).

Sodium alginate has commonly been used to produce gel beads on a millimetre scale for cell encapsulation, due to their high degree of biocompatibility and mild gelling conditions in the presence of divalent cations (Lee and Mooney, 2012; Rowley and Mooney, 2002; Wang et al., 2005; Lemoine et al., 1998). Stiffness of alginate can be altered by changing either the viscosity of the alginate or the alginate/crosslinker concentration (Andersen et al., 2014; Chan et al., 2011). Using a water-in-oil emulsion method, aqueous sodium alginate was dripped into an oil bath and continuously stirred for 30 min to allow the formation of alginate beads. The addition of calcium chloride induced rapid gelation of the alginate beads, which were collected by centrifugation after extensive washing with isopropanol (Fig. 2A).

Next, we exposed alginate beads synthesised from low and intermediate viscosity alginates to poly-L-lysine (PLL). Electrostatic interactions between the negatively charged alginate bead surface and the positively charged PLL residues induces the formation of a polyanionic/cationic coat at the bead surface (Thu et al., 1996) (Fig. 2B). The formation of a PLL coat has been shown to enhance the mechanical properties of alginate (Bhujbal et al., 2014; Kleinberger et al., 2013), and by exposing beads to differing concentrations of PLL, beads were produced across a wide range of rigidities. Using AFM to quantify bead rigidities, three distinct bead batches were produced: 9±7 (soft), 34±15 (medium) and 254±199 kPa (stiff) targets (Fig. 2C). Measuring the bead diameters revealed that, although softer beads had slightly larger bead diameters, >70% of bead diameters were ≤20 µm (Fig. 2D), similar to cells commonly used as immune target cells, such as K562 (17 µm) and Raji (5-8 µm).

To activate NK cells, PLL-coated alginate beads were coated with anti-LFA-1 and anti-NKp30 IgG1κ mAb. To ensure mAb levels across the bead batches were similar, beads were coated with anti-NKp30 mAb directly conjugated to an AF647 fluorophore and their fluorescent intensity was compared across the bead batches. Using imaging flow cytometry, fluorescence was detected on the surface of all three bead batches (Fig. 2E; Fig. S2A). The 9 kPa beads were incubated with mAb at five-fold greater concentration to match levels of binding to beads of 34 kPa and 254 kPa stiffness, at which point mean fluorescence intensity (MFI) levels were similar across the three different batches (Fig. 2E-G; Fig. S2B). The density of coating antibody was overlapping between stiffness batches (Fig. S2C). A minority of low stiffness beads exhibited internal fluorescence and stained particularly brightly. This small fraction of abnormal beads was excluded from our analysis in all imaging experiments. Fig. S2D shows examples of such beads exhibiting internal staining, and the soft bead images actually analysed are shown in Fig. S2E for comparison.

### Target stiffness directly impacts NK cell degranulation efficiency

**Fig. 3.**
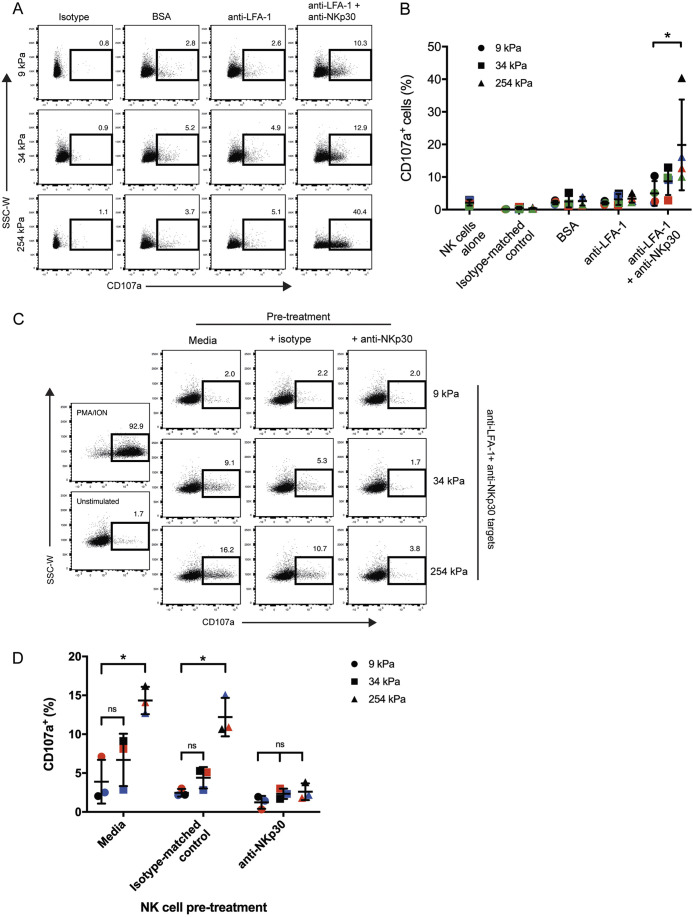
**NKp30-dependent NK cell degranulation is sensitive to changes in target stiffness.** (A) Primary NK cells were mixed at a 1:1 ratio with 9, 34 and 254 kPa beads coated with either BSA, anti-LFA-1 mAb alone or in combination with anti-NKp30 mAb. Conjugates were left for 4 h, and NK cells were assessed for surface expression of CD107a by flow cytometry. Dot plots shown from one donor are representative of four independent experiments. (B) Quantification of CD107a^+^ cell frequencies. Each different coloured data point represents an individual donor (*n*=4). (C) To block the NKp30 receptor, primary NK cells were pre-treated with either an IgG1 isotype-matched control mAb, anti-NKp30 mAb or no mAb (medium) for 1 h at 37°C. NK cells were subsequently mixed with beads coated with anti-LFA-1 mAb or anti-LFA-1 and anti-NKp30 mAb at a 1:1 ratio, and left for 4 h at 37°C. PMA and ionomycin were added to cells in a separate well after 3 h, and incubated for 1 h. Cells were subsequently analysed for CD107a surface expression by flow cytometry. Dot plots show live single cells in conjugate with beads coated with anti-LFA-1 and anti-NKp30 mAbs; representative from one donor out of three analysed. (D) Quantification of CD107a^+^ cell frequencies of NK cells in conjugate with beads coated with anti-LFA-1 and anti-NKp30 mAbs. Each different coloured data point represents an individual donor (*n*=3). *P<0.05; ns, not significant (Friedman test with Dunn's multiple comparisons).

Next, we set out to determine whether engaging targets of varying rigidities impacts NK cell degranulation. Primary NK cells were mixed with 9, 34 and 254 kPa (soft, medium and stiff targets, respectively) beads coated with either bovine serum albumin (BSA), anti-LFA-1 mAb alone or anti-LFA-1 with anti-NKp30 mAb. In response to beads coated with BSA or anti-LFA-1 alone, there was little, if any, staining of CD107a above that of unstimulated NK cells (incubated in medium alone), confirming minimal NK cell degranulation with BSA or anti-LFA-1 coated beads (Fig. 3A,B). However, upon co-incubation with beads coated with anti-LFA-1 and anti-NKp30 mAbs, the proportion of NK cells which stained with CD107a, and therefore degranulated, increased as the stiffness of the target increased; 5.0±3.8%, 8.7±4.2% and 19.8±14.0% for soft, medium and stiff targets, respectively (Fig. 3B).

Although these results suggest that stiffness increases degranulation, it could not be ruled out that these observations were due to the thicker PLL membrane on the medium and stiff beads, or some other non-specific mechanism of activation. To confirm that the differences in degranulation responses were due to the altered substrate rigidity, we blocked the NKp30 receptor on NK cells using anti-NKp30 mAb before the start of the degranulation assay. This should abrogate degranulation in the absence of any other stimulation. Indeed, blocking the NKp30 receptor completely abolished degranulation when incubated with soft, medium and stiff beads, demonstrating degranulation was occurring via NKp30 (Fig. 3C,D). Altogether, these data establish that increasing the tension of the target substrate results in enhanced degranulation triggered via NKp30.

### The NK cell spreading response is sensitive to target stiffness

The NK cell cytolytic response is triggered in multiple stages (Orange, 2008) and thus, we next set out to assess at which stage the NK cell response was impaired against soft targets. Maturation of the synapse is dependent on an initial spreading of the NK cell against the target interface, mediated and sustained by actin polymerisation at the cell periphery. Thus, we tested whether or not actin polymerisation at the synapse was impacted by changes in target stiffness.

**Fig. 4.**
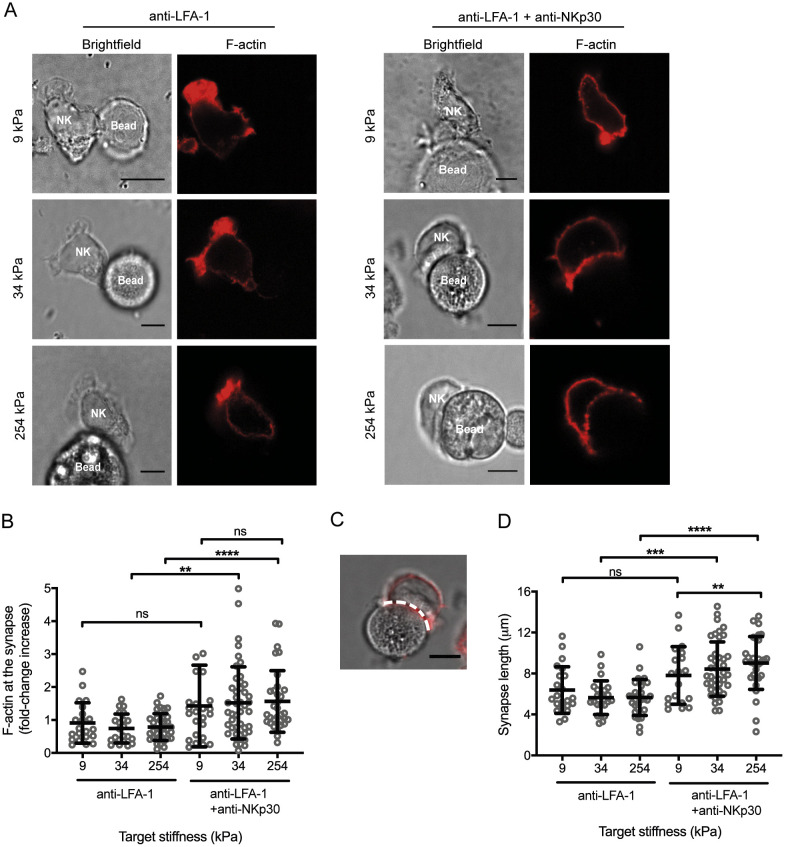
**Assembly of F-actin at the NK cell synapse is moderately impacted by changes in target stiffness.** (A) Primary NK cells were mixed with 9, 34 and 254 kPa beads coated with anti-LFA-1 mAb alone or in combination with anti-NKp30 mAb, and incubated at 37°C for 20 min. Conjugates were fixed, permeabilised and stained with phalloidin-AF647 to stain F-actin (shown in red). Representative images of 5-15 cells, from three donors analysed in independent experiments. (B) Quantification of F-actin present at the synapse. The amount of F-actin present was quantified by measuring the mean fluorescence intensity of F-actin at the synapse and dividing it by the mean fluorescence intensity of an equivalent-sized region of the membrane at the back of the cell. (C) When quantifying synapse length, the image of cellular fluorescence was overlaid over the corresponding bright-field image and a line drawn over the cell synapse (white dotted line). (D) Quantification of synapse lengths as described in B. Data points on the graphs represent individual conjugates (5-15 conjugates per donor) pooled from independent donors (*n*=3). Data are mean±s.d. **P<0.01; ***P<0.001; ****P<0.0001; ns, not significant (one-way ANOVA with Tukey's multiple comparison's test). Scale bars in all images: 10 μm.

Primary NK cells were mixed with soft, medium and stiff bead targets for 20 min, fixed and F-actin stained with phalloidin-AF647 (Fig. 4A). To assess the amount of F-actin at the synapse, the fluorescence intensity of phalloidin staining at a cell/bead interface was compared to a region of the same size at the back of the NK cell. Low target stiffness did not entirely stop accumulation of F-actin at the synapse (Fig. 4A,B). However, for medium and stiff targets, the mean increase in F-actin at the synapse was much more pronounced, doubling when anti-NKp30 mAb was present (with a fold-change intensity increase from 0.7±0.4 to 1.5±1.0 and from 0.8±0.4 to 1.6±0.9 for 34 kPa and 254 kPa targets, respectively, Fig. 4B). Additionally, the proportion of NK cells in conjugates was higher against 254 kPa targets (Fig. S3A).

To assess NK cell spreading against the different targets, the length of each synapse was measured (example shown in Fig. 4C). In conjugates with NKp30 ligated, there was a strong increase in NK cell spreading against stiff targets, more than for soft targets (9.0±0.5 µm versus 7.8±0.6 µm, Fig. 4D).

### Polarisation of the MTOC towards the immune synapse is dependent on the mechanical properties of the target interface

Triggering of an activating IS results in rapid polarisation of perforin-rich lytic granules towards the cell/target interface, which is dependent on the polarisation of the MTOC. Thus, we next assessed whether or not the MTOC polarised similarly against targets of different stiffness. Primary NK cells were incubated with soft, medium and stiff targets for 20 min, fixed, permeabilised and stained for F-actin, MTOC and perforin-rich granules.

**Fig. 5.**
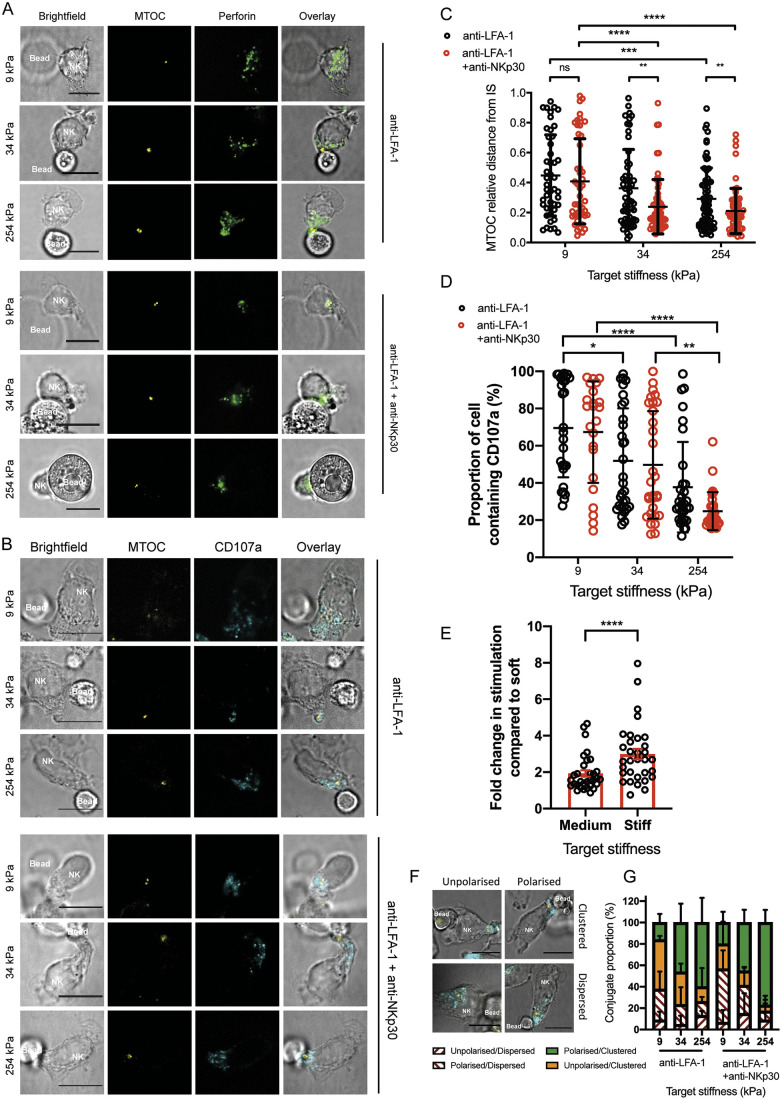
**NKp30 ligation on soft targets fails to induce MTOC and granule polarisation towards the synapse membrane.** (A) Primary NK cells were mixed at a 1:1 ratio with 9, 34 and 254 kPa beads coated with either anti-LFA-1 mAb or anti-LFA-1 and anti-NKp30 mAb. Cells were incubated at 37°C for 20 min, fixed and stained with anti-perforin-AF488 (shown in green) and anti-pericentrin mAbs, followed by anti-IgG1 k-AF568 mAb (shown in yellow) and imaged using confocal microscopy. (B) Using the same setup, cells were stained with anti-LAMP1-AF647 (shown in cyan) and imaged using confocal microscopy to determine the polarisation of cytotoxic granules to the synapse. (C) Polarisation of the MTOC was quantified by dividing the distance of the interface to the MTOC by the length of the NK cell. Thus, a value of 0 implies complete polarisation to the synapse. Data pooled from six donors; 5-15 cells imaged per donor. (D) Quantification of the proportion of the cell containing granules. The length of the cell containing granules was divided by the total length of the cell. (E) Summary data showing fold change in stimulation across all donors. Across all metrics, the NK responses to medium and stiff targets were divided by the response to soft targets and expressed as fold changes (*n*=32, ****P<0.0001). Data are mean±s.e.m. (F) Conjugates were scored on granule clustering and MTOC polarisation. Granules were scored by eye on the degree of clustering around the MTOC. All conjugates in which the degree of clustering was unclear were discarded (<5%). Polarisation of the MTOC was quantified by dividing the distance of the interface to the MTOC by the length of the NK cell. Values <0.3 were categorised as polarised. (G) Quantification of granule clustering and MTOC polarisation. The mean percentage of unpolarised/dispersed (white and red), polarised/dispersed (white and red), unpolarised/clustered (orange) and polarised/clustered (green) conjugates were plotted. Each bar represents the mean of three donors (5-15 cells per donor). Data are mean±s.e.m. (C-E) or mean±s.d. (G). *P<0.05; **P<0.01; ***P<0.001; ****P<0.0001; ns, not significant (one-way ANOVA with Kruskal–Wallis test for multiple comparisons). Scale bars: 10 μm.

NK cells that formed conjugates with 9 kPa targets coated in anti-LFA-1 mAb alone were significantly less polarised than 254 kPa targets (0.45±0.27 compared to 0.29±0.21 for the relative distances between the IS and MTOC, Fig. 5A,C). With NK cell activation triggered via anti-NKp30, 9 kPa targets failed to induce MTOC polarisation as strongly as 254 kPa targets (0.21±0.15 compared to 0.41±0.29). Thus, target stiffness plays an important role in triggering polarisation of the MTOC towards the IS. We found no significant differences over time, indicating that the observed differences in MTOC polarisation between the targets of different stiffness did not arise through slower activation (Fig. S3B). As different antibodies can engage LFA-1 in different ways (Fan et al., 2019), we tested an alternative anti-LFA-1 mAb (HI111). Similarly with this antibody, MTOC polarisation was far more pronounced against stiff targets (Fig. S3C). To test whether or not activation by soft or stiff beads could be affected by ligand density, we also assessed the degree of MTOC polarisation when (1) 9 kPa beads were coated in especially high concentrations of anti-NKp30 and (2) 254 kPa beads were coated in low concentrations of anti-NKp30. Even a high ligand density on soft surfaces did not trigger cellular activation. Moreover, weak ligation by stiff targets still induced NK cell polarisation. This further establishes the importance of target stiffness irrespective of ligand dose (Fig. S3D). Moreover, the observed trends were robust to analysing F-actin accumulation and granule polarisation in three dimensions (Fig. S3E).

Before polarisation, perforin-rich granules translocate along microtubules and cluster around the MTOC (Mentlik et al., 2010; Orange, 2008). By staining CD107a and the MTOC simultaneously we tested whether or not granule coalescence and polarisation were affected by target stiffness (Fig. 5B). By dividing the distance from the IS to the furthest granule by the length of the cell, we calculated the percentage of the cell occupied by granules (Fig. 5D). The granules were typically spread throughout the cell in conjugates with 9 kPa targets, occupying 70±27% and 67±27% of the cell, for beads coated with anti-LFA-1 alone and with NKp30 mAb, respectively. There was a marked decrease in granule spread when NK cells were conjugated to 254 kPa targets, with the proportion of the cell containing granules being 38±24% and 25±10%. Fig. 5E summarises the fold change in stimulation for all donors used in the paper, across all assays. Specifically, the fold change in each response of the donor against the medium and high stiffness targets is shown in comparison to the response of the donor to low stiffness targets. Thus, across 32 donors and multiple types of assay, NK cells were better activated by stiffer targets.

Granule coalescence and MTOC polarisation together are critical steps in cytolysis. Analysing the two parameters together reduces uncertainty that the MTOC is classed as polarised or that the granules clustered by chance. We scored all images by eye as polarised/unpolarised (MTOC) and clustered/dispersed (granules) and plotted the four frequencies for beads coated in anti-LFA-1 alone and with anti-NKp30 (Fig. 5F,G). As granule coalescence occurs before MTOC polarisation (Mace et al., 2014), cells in which granules were dispersed (white bars with red lines) essentially indicated a lack of reactivity (whether or not the MTOC shows an apparent polarisation). These constituted the majority of conjugates with 9 kPa beads coated in NKp30 mAb (7±12% and 50±17% for conjugates with polarised and unpolarised MTOC, respectively). Conjugates with an unpolarised MTOC but clustered granules (amber bars) represented 23±21% of conjugates overall. Only 20±10% of conjugates were cytolytic, with clustered granules and polarised MTOC (green bars). In contrast, 254 kPa targets coated in NKp30 mAb far more effectively triggered these steps in cytolytic conjugate formation, with 77±12% of conjugates having clustered granules and a polarised MTOC. Blocking NKp30 abrogated MTOC polarisation against beads coated in LFA-1 and NKp30 mAb, and reduced MTOC polarisation against beads coated in LFA-1 mAb alone (Fig. S4). It is not entirely clear as to why anti-NKp30 mAb blocking would affect polarisation triggered via LFA-1 alone, but perhaps this relates to the nanoscale proximities of activating receptors and integrins on the cell surface such that blocking one impacts the accessibility of the other.

### Synapse stability is sensitive to target stiffness

Target stiffness influences several steps in NK cell cytotoxicity, with stiff targets inducing slightly more NK cell spreading, and increased polarisation of the MTOC and perforin granules. T-cell kinapses denote asymmetrical unstable interactions between T cells and targets in which the lymphocyte is not sufficiently activated to halt motility (Dustin, 2008). Using beads coated in anti-LFA-1 mAb with or without anti-NKp30, we set out to characterise the proportion of conjugates in which the NK cell phenotype indicated motility, with the focus of F-actin at the leading edge lamellipodium separate from the IS.

**Fig. 6.**
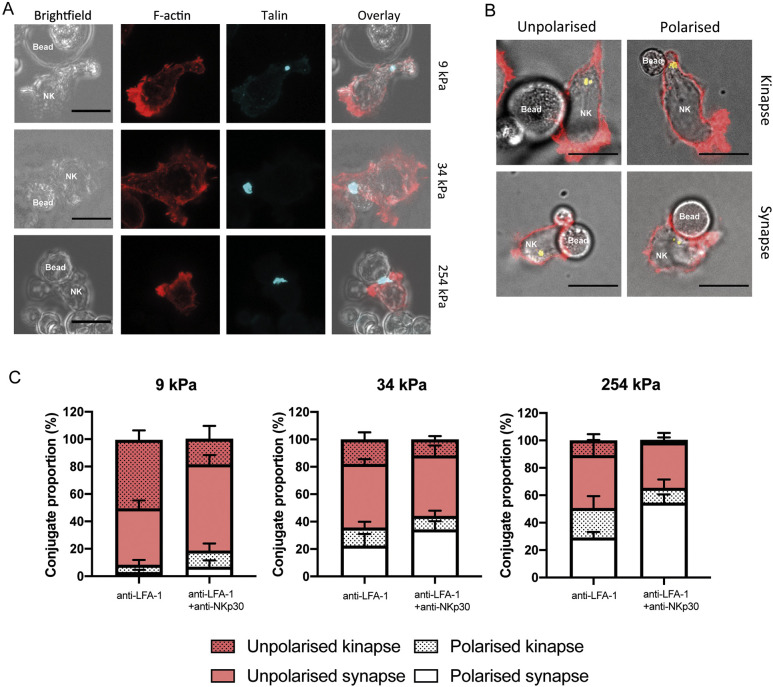
**Target stiffness determines stability of synapse.** (A) Primary NK cells were mixed at a 1:1 ratio with 9, 34 and 254 kPa beads coated in anti-LFA-1 and anti-NKp30 mAbs, and left to form conjugates for 20 min. After fixing, conjugates were stained with anti-talin mAb with goat anti-mouse IgG (H+L) AF568 secondary antibody (shown in cyan) and phalloidin-AF647, and then imaged using confocal microscopy. Images show talin accumulation at the point of contact between NK cells and beads, representative of 3-5 cells from three independent donors. (B) Conjugates were scored on MTOC polarisation and localisation of F-actin. Polarisation of the MTOC was quantified by dividing the distance of the interface to the MTOC by the length of the NK cell. Values of <0.3 were categorised as polarised. NK cells were denoted as forming a kinapse when F-actin was asymmetrically distributed relative to the line bisecting the point of contact. All conjugates in which the degree of symmetry was unclear were discarded (<5%). (C) Quantification of the percentage of synapse/kinapse formation against beads of different stiffnesses, subdivided based on MTOC polarisation. Data are mean±s.e.m. [mean of seven donors (5-15 conjugates per donor)]. Scale bars: 10 μm.

Talin polarisation to the contact between lymphocyte and targets is dependent on LFA-1 ligation, and is a precursor to both synapse and kinapse formation (Smith et al., 2005). Importantly, against all bead conditions we observed a similar accumulation of talin in focal zones (Fig. 6A; Fig. S5), similar to those described previously against ICAM-1 coated polystyrene beads (Mace et al., 2009). This indicated that the NK cells were interacting with, rather than bypassing, the beads, irrespective of their stiffness.

We categorised each conjugate, according to the positions of the MTOC and organisation of F-actin within the NK cell, into four groups: unpolarised kinapse; polarised kinapse; unpolarised synapse; and polarised synapse (Fig. 6B). Conjugates were denoted kinapses in which the distribution of F-actin was clearly asymmetrical relative to the line perpendicular to the bead/cell interface. All conjugates in which the degree of symmetry was unclear were discarded. Cells with an MTOC ratio of <0.3 were considered polarised. The proportions of each type of interaction (unpolarised/polarised and synapse/kinapse) were determined for interactions between NK cells and 9 kPa, 34 kPa and 254 kPa targets (Fig. 6C). Against 254 kPa targets, synapses outnumbered kinapses in NK cell interactions that were both polarised and unpolarised, indicating that these targets provided a threshold stiffness for the establishment of symmetrical synapses. Against 34 kPa targets coated in anti-NKp30, the percentage of unstable kinapses that resulted in either polarised or unpolarised NK cells constituted 10±10% and 12±6% of interactions, respectively. Against soft 9 kPa targets, interactions were predominantly either unpolarised and/or kinapses. Analogous data were obtained when NK cells were stimulated via the antibody-dependent cellular cytotoxicity pathway through CD16 ligation (Fig. S6). Thus, a soft target surface does still allow interaction with an NK cell, as evidenced by talin recruitment, but does not meet a threshold required for symmetrical synapse formation. Taken together these results demonstrate that target mechanical properties and receptor ligation both contribute to the formation of a stable symmetrical synapse.

## DISCUSSION

Diseased tissue often presents with altered extracellular matrix protein compositions giving rise to changed stiffness of both the matrix and cells present (Engler et al., 2006; Cross et al., 2007; Liu et al., 2015; Paszek et al., 2005). There have been several studies of how differences in force impact on TCR signalling (Simonson et al., 2012; O'Connor et al., 2012; Judokusumo et al., 2012; Springer and Dustin, 2012; Liu et al., 2021) and T-cell function (Saitakis et al., 2017; Ricker et al., 2016; O'Connor et al., 2012). Despite their role in tumour surveillance, and the established link between mechanical changes and oncogenesis, the effect of target stiffness change on NK cell effector functions has been far less studied.

Here, we set out to investigate how altering the stiffness of the target interface influenced NK cell cytotoxic functions. Polyacrylamide hydrogels coated with mAb against the activating receptor NKp30 and the integrin LFA-1 were used to mimic an activating target interface. We establish that the pro-inflammatory cytokine IFNγ, as well as granulysin, FasL and granzymes A and B, are secreted by human NK cells in response to LFA-1 and NKp30 ligation, with increasing secretion observed on stiffer substrates. NK cells on soft substrates activated by the addition of PMA and ionomycin degranulated to the same extent as NK cells on the stiffest substrates, implying that NK cells on soft substrates are not incapacitated, but rather receptor-mediated activation of NK cells is mechanically sensitive.

To our knowledge, all previous evidence of varied cellular cytotoxic responses to mechanical stiffness has relied on two-dimensional substrates. We present a novel methodology for using PLL-coated alginate beads as surrogate targets to study single-cell responses in three dimensions. This approach establishes that the mechanism of stable IS formation is optimised for stiff targets.

A small number of ill-formed 9 kPa beads were present in our production process, which exhibited some level of internal staining. But these cannot account for differences in stimulation for a number of reasons. Aberrant 9 kPa beads with internal staining were excluded from experiments based on image analysis. Moreover, beads of 34 kPa and 254 kPa did not exhibit any level of internal stain, yet there were significant differences between the ability of these beads to stimulate NK cells. The density of protein staining was consistent across different bead types. And perhaps most importantly, if the level of antibody coating was changed dramatically, soft beads with high staining were impaired in their ability to activate NK cells, whereas hard beads with low staining still activated NK cells effectively.

Mechanical force and ligand binding can be functionally intertwined (Comrie et al., 2015), and a lack of opposing force with soft targets perhaps fails to engage the molecular clutch upon LFA-1 engagement. Additionally, soft targets are prone to ruffling and may provide insufficient traction forces for the outward growth of the immune synapse. A similar phenomenon was seen when studying fibroblast movement over rubber (Harris et al., 1980). Greater forces produced by stiffer targets likely allow faster and more sustained actin retrograde flow, with symmetrical spreading of the NK cell around the target. Against soft targets, random slippage events occur during synapse formation (Matalon et al., 2018) and the contractile forces of molecular motors dissipate, which can cause actin filaments to slide (Gupta et al., 2016).

The tension of the target interface is also likely to be important in aiding myosin-generated forces, which serve to remodel the F-actin meshwork at the IS, allowing granules to translocate to the synaptic membrane (Carisey et al., 2018). Inhibiting myosin motor protein activity, either by RNAi-mediated knockdown or by chemical inhibitors, blocks granule fusion to the NK cell membrane (Sanborn et al., 2009; Andzelm et al., 2007). Total internal reflection fluorescence imaging of lytic granules at the IS in YTS cells spread on CD28-coated slides showed that myosin inhibitors diminished the penetration of granules into F-actin at the IS (Sanborn et al., 2009). Moreover, inhibition of myosin activity led to an increased density of fibres, resulting in fewer granule-permissive channels at the synapse, despite retention of the overall actin-mesh architecture (Carisey et al., 2018). Microtubules play a crucial role in mediating MTOC and perforin translocation to the synapse (Huse, 2012). Of all the effects of varied target stiffness, which we tested here, the strongest correlate was MTOC polarisation.

The extent to which signalling through NKp30 is directly affected by target stiffness remains to be established. NKp30 signals via an associated CD3ζ chain, which induces the recruitment of the tyrosine kinases SYK and ZAP70. These kinases in turn phosphorylate transmembrane adaptor molecules, such as LAT, leading to activation of PLC-γ1 and the Vav family of proteins (Watzl and Long, 2010; Watzl et al., 2014). T cells plated on activating soft polyacrylamide substrates were devoid of phosphorylated SYK and ZAP70 kinases within the cell centre even by 30 min post-seeding. In contrast, stiffer substrates showed rapid accumulation of phosphorylated ZAP70 and SYK already within 2 min post-seeding (Judokusumo et al., 2012). Thus, the low percentage of NK cells degranulating with soft targets may result from poor recruitment of phosphorylated ZAP70 to the NK cell synapse (Matalon et al., 2018).

Several studies have documented a reduction in stiffness during early apoptosis as a result of cytoskeletal degradation (Pelling et al., 2009; Su et al., 2019; Schulze et al., 2009). A lack of reactivity to such soft targets may have evolved to conserve energy and/or cellular components that would otherwise be wasted in an attempt to kill targets that were already apoptotic. Metastatic cancer cells are one critical exception in which a lower stiffness may signal danger. Invasive cells, such as SKOV3 and HEY, display softer mechanical characteristics, allowing the shape changes required to undergo metastasis (Swaminathan et al., 2011). On account of their reduction in stiffness, NK cells may fail to respond to these softer targets. Glioma cells are another example in which disease progression is correlated with a decrease in cellular stiffness (Pepin et al., 2018). Intriguingly, glioblastoma cells evade T-cell killing by locally preventing IS formation, with interactions characterised by kinapse dynamics (Díaz et al., 2018). There are settings in which it may be preferential for an NK cell not to form a full IS, with a kinapse-like mode of interaction allowing deeper penetration into solid tumours (Deguine et al., 2010).

Overall, our data establish the importance of target stiffness in regulating NK cell cytotoxicity. We have revealed that soft targets represent a blind spot in NK cell killing, which suggests that acquisition of mechanical softness by diseased cells may be a novel immune evasion mechanism.

## MATERIALS AND METHODS

### Isolation of human primary NK cells

Primary human NK cells were isolated from the peripheral blood of healthy donors from the National Blood Service under ethics licence REC 05/Q0401/108 (University of Manchester, UK). Peripheral blood mononuclear cells (PBMCs) from healthy donors were obtained by separation on a Ficoll-Paque gradient. NK cells were acquired from PBMCs via negative selection using an NK cell isolation kit (Miltenyi Biotec). NK cells were maintained in Dulbecco's Modified Eagle Medium, 30% F12 Ham, 10% human serum, 1% non-essential amino acids, 1 mM sodium pyruvate (Sigma-Aldrich), 2 mM L-glutamine, 50 U/ml penicillin streptomycin and 50 µM 2-mercaptoethanol (Gibco). NK cells were stimulated with rhIL-2 (200 U/ml; Roche) and rested for 6 days before experiments. Cells were kept at 37°C in a 5% CO_2_ atmosphere.

### Preparation and synthesis of polyacrylamide hydrogels

To prepare glass surfaces for gel binding, 3 µl bind-silane (GE Healthcare), 47 µl glacial acetic acid and 950 µl ethanol (Fisher Scientific) were mixed together, and 200 µl of this mix was pipetted onto glass-bottomed dishes (No. 0, 14 mm diameter; MatTek). After 3 min, dishes were rinsed with ethanol and left to dry at room temperature. Separately, to clean glass coverslips (No. 0, 13 mm diameter), they were initially sonicated in acetone for 15 min, rinsed twice with water and rocked for 1 h in NaOH (1 M). Coverslips were washed extensively with water and stored in 70% ethanol until use.

Hydrogels were synthesised using ProtoGel (National Diagnostics) adapted from a method described by Wang and Pelham (1998). ProtoGel, a stabilised mixture of 30/0.8% (w/v) acrylamide/bis-acrylamide solution was mixed with PBS (90, 80 and 50% of the final volume) to synthesise gels with Young's moduli of 1, 22 and 142 kPa, respectively. The ProtoGel mixture was degassed in a vacuum chamber whereupon 100 mg/ml ammonium persulfate (Sigma-Aldrich; 1/100) and N,N,N′,N′-tetramethylethane-1,2-diamine (TEMED; Sigma-Aldrich; 1/1000) were added to the solution. The solution was added onto a silianised glass bottom dish and a clean glass coverslip was immediately placed on top. Post-gelation, the dish was flushed with PBS and the top coverslip was removed with forceps. The gels were washed twice with PBS to remove unbound acrylamide and stored at 4°C.

### Quantification of hydrogel stiffness

The hydrogel stiffness was characterised by the reduced Young's Modulus (*E*). Stiffness values were obtained from generating force curves gathered using a Bruker Bioscope Catalyst AFM with a Nanoscope V controller (Bruker) mounted on a Nikon Eclipse Ti-I optical microscope operating under the NanoScope controller software (v9.15) (Bruker). The gels were probed using a silicon nitride spherically tipped cantilever [Windsor Scientific; nominal spring constant (*k*) of 0.3 N/m; 2.5 µm nominal radius]. The spring constant was calibrated using the thermal oscillation tuning method (Lübbe et al., 2013). For the AFM setup, the cantilever was aligned over the sample using a camera (Zeiss Axiocam MRc) attached to the microscope, and the relationship between the photodiode signal and cantilever deflection (deflection sensitivity) was obtained from force curves generated on glass.

The local reduced modulus was determined for five different locations on the gel surface in a 1×1 µm^2^ region, indented (100 nm depth) at a frequency of 1 Hz with lateral spacing of 0.1 µm. Analysis was carried out using force curve analysis software (NanoScope analysis v1.40; Bruker) whereby a baseline correction was applied to each curve before a force fit was applied using the Hertzian (spherical) model (Lin et al., 2007) and a maximum force fit of 70%. Any force values falling more than 2 s.d. away from the mean value for a given area were discarded to account for failed indents.

### Functionalisation of the gel surface

Gels were washed with 20 mM HEPES, and any residual liquid was aspirated from the surface. A layer of 1 mg/ml of the heterobifunctional crosslinker sulfo-sanpah (Thermo Fisher Scientific) was pipetted on the gel surface and the gels irradiated under UV light (254 nm) for 10 min. The gels were washed three times with 20 mM HEPES then incubated in the dark for 18 h at 4°C with mAbs. These were diluted in PBS at the following concentrations: 30/20/20 µg/ml for 1, 22 and 142 kPa gels, respectively, of both NKp30 mAb (P30-15, BioLegend) and/or LFA-1 mAb (TS2/4, BioLegend). For gels coated with anti-LFA-1 mAb, a matched non-stimulatory murine isotype control was used to ensure protein densities were similar across the different conditions (IgG1 κ, BioLegend). Gels were washed twice with PBS and stored at 4°C for up to 48 h.

To examine anti-NKp30 mAb densities on the surface, gels were blocked with 3% BSA/PBS for 1 h at room temperature and then incubated with goat anti-mouse IgG1-AF488 H&L secondary antibody (A11001; Invitrogen) for 1 h at room temperature. Gel surfaces were imaged using confocal microscopy (Leica SP8, 63×/1.20 NA oil-immersion objective) with fluorescent beads (592 nm FluoSpheres, 580/605; 1/1000 dilution in PBS; Thermo Fisher Scientific) placed on the surface before imaging to determine the correct focal plane. The fluorescence intensity from each gel was obtained from the average of the MFI of three different areas of the gel surface.

### Gel microbead synthesis

Two different alginates were used in this study. Intermediate viscosity alginate (75-150 kDa; 40% guluronic content and 60% mannuronic content, Pronova SLM20, Novamatrix) was used to synthesise 9 kPa beads and low viscosity alginate (12-80 kDa; 40% guluronic content and 60% mannuronic content, A0682, Sigma-Aldrich) was used to synthesise 34 kPa and 254 kPa beads. Alginate was dissolved in sterile water (2% or 5% w/v for 9 kPa or 34/254 kPa beads, respectively) and pipetted dropwise into a sunflower oil bath containing 0.5% Tween 20 (Sigma-Aldrich). The final ratio of alginate/oil was 1:4 (2.5 ml alginate in 10 ml oil/Tween 20). The solution was stirred using a magnetic stirrer (1500 rpm) for 30 min to allow the alginate droplets to form stable beads. Subsequently, calcium chloride dissolved in sterile water (0.25 or 10% w/v for soft and medium/stiff beads, respectively) was slowly pipetted dropwise (4 ml) into the alginate/oil mixture and then incubated for 1 h at room temperature to gel.

For 34/254 kPa beads, post-gelation, 10 ml of isopropanol (Fisher) was added to the stirring mixture, which was then incubated for 10 min whereupon the solution was transferred to a 50 ml Falcon tube. For 9 kPa beads, after 1 h the bead mixture was immediately transferred to a 50 ml Falcon tube. Beads were spun down at 1500 ***g*** for 10 min and the supernatant removed. The beads were spun down a further four times, using fresh isopropanol each time (40 ml). Beads were then washed twice and resuspended in 100 ml sterile water, and filtered through a 40 µm cell strainer (Fisher) to remove beads or debris >40 µm. The flow through was spun down at 1500 ***g*** for 10 min and the beads were resuspended at 2×10^6^/ml in sterile water.

### Poly-L-lysine and protein coating

PLL (MW 15-30 kDa; P7890; Sigma-Aldrich) was dissolved in sterile water (0.1% w/v) and stored in sterile conditions at 4°C for up to 6 months. Beads (2×10^6^/ml) were incubated in differing dilutions of PLL (0.0005, 0.002 and 0.005% for 9, 34 and 254 kPa beads, respectively) in a total volume of 10 ml. The 9 kPa beads were incubated with PLL for 2 min while being continuously rocked (35 rpm), and 34/254 kPa beads were vortexed continuously for 10 min for optimal coating. Post-incubation with PLL, beads were spun down (1500 ***g***) for 5 min, and the supernatant was removed and washed twice with PBS.

PLL-coated beads were counted using a haemocytometer and 0.4×10^6^/ml beads were transferred to 1.5 ml Eppendorf tubes in 200 µl PBS. Anti-NKp30 mAb (P30-15, BioLegend), anti-CD16 mAb (3G8, BioLegend) and/or anti-LFA-1 mAb (TS2/4 or HI111 where specified, BioLegend) were added as indicated (each at a total concentration of 50/10/10 µg/ml for 9, 34 and 254 kPa beads, respectively) and left for 1 h while being continuously shaken at room temperature. For beads coated with anti-LFA-1 mAb only, a matched non-stimulatory murine isotype control was used in place of anti-NKp30 to ensure protein densities were similar. After 1 h, beads were spun down (2000 ***g***) and washed twice with 1 ml PBS, and blocked with 3% BSA/PBS for 30 min while being continuously shaken. Beads were washed with 1 ml PBS before being added to cells. To test the amount of anti-NKp30 mAb bound to the bead surface, beads were incubated for 1 h with anti-NKp30-AF647 (labelled with AF647 NHS ester, Thermo Fisher Scientific) at room temperature. The geometric mean fluorescence intensity (gMFI) for each bead batch was determined using flow cytometry (Imagestream, Amnis).

### Characterising bead stiffness

Bead stiffness was characterised using the same system as per hydrogel stiffness quantification. Beads were immobilised to prevent bead movement during force curve acquisition. Briefly, 1×10^5^/ml PLL-coated beads were extruded through a 10 µm membrane filter (13 mm diameter; Millipore) using a 1 ml syringe. The membrane filter was subsequently fixed to a glass slide and submerged in PBS. Individually trapped beads within the membrane pores were found using a camera (Zeiss Axiocam MRc) attached to the microscope.

Force curves were obtained, for beads submerged in PBS, via an indentation made with a borosilicate glass colloidal probe mounted on a cantilever [CP-CONT-BSG-A, sQUBE; nominal spring constant (*k*) of 0.3 N/m; 2.5 µm nominal radius]. The spring constant was calibrated using the thermal oscillation tuning method (Lübbe et al., 2013). The local reduced modulus for each bead was determined by indenting ten different points on each bead.

### Cytokine bead array

To stimulate cytokine secretions, 2×10^5^ NK cells were incubated on flat polyacrylamide substrates coated with anti-LFA-1 or anti-LFA-1 plus anti-NKp30 mAb for 24 h. The supernatants were subsequently collected and stored at −20°C for further analysis. Cytokines in the supernatant were detected using a cytokine bead array kit (CD8/NK cell LEGENDplex kit; BioLegend) according to the manufacturer's instructions. The samples were analysed by flow cytometry (FACSVerse; BD) and data were analysed using flow cytometry analysis software (LEGENDplex software v8.0; BioLegend).

### Degranulation assays

For measuring NK cell degranulation, 5×10^5^ NK cells were mixed with 5×10^5^ beads in a total volume of 100 µl medium (1:1 E:T ratio) in a 96 U-bottomed plate. Alternatively, for examining degranulation on flat polyacrylamide substrates, 2×10^5^ cells were added in 200 µl to the gel surface. Cells were incubated on hydrogels or with beads in the presence of anti-CD107a-PE (H4A3, BD Biosciences) and protein transport inhibitors brefeldin (GolgiPlug; 1/1000 dilution, BD Biosciences) and monensin (1/1000 dilution, BioLegend) at 37°C for 4 h. Together, these drugs allow accumulation of CD107a within the cell, ensuring the signal is not lost through the recycling of this protein. Dead cells were excluded using a viability dye (Zombie NIR; BioLegend), and cells were stained with anti-CD107a-PE or isotype-matched controls. PMA and ionomycin (both at 1 µg/ml; Sigma-Aldrich) were used to stimulate NK cell degranulation via soluble stimulation, and were added to a separate well of NK cells 1 h before the end of the assay. Where indicated, the blocking of NKp30 was carried out by incubating NK cells (1×10^6^/ml) with anti-NKp30 mAb (P30-15; 20 µg/ml), or an isotype-matched control mAb for comparison, for 1 h at 37°C before the start. These cells were washed in fresh medium before the start of the degranulation assay.

### Imaging NK cell–bead interactions

To prepare NK cell-bead conjugates, protein-coated beads were spun down at 2000 ***g*** for 5 min and resuspended in human serum-free medium. Beads were plated out into glass-bottomed wells (Labteks no.1.5; Nunc; 4×10^5^ soft beads per well and 2×10^5^ medium/stiff beads per well) pre-coated with 10 µg/ml fibronectin (F0985, Sigma-Aldrich). Soft beads were plated at a higher concentration as they were more difficult to locate when imaging. Beads were allowed to settle for 1 h at 37°C. NK cells were spun down at 300 ***g*** for 5 min and the supernatant was removed. The cell pellet was resuspended in medium with 10% fetal bovine serum, and 2×10^5^ cells were added into each well. Conjugates were left to form for 20 min, then fixed by the addition of 4% paraformaldehyde/PBS for 20 min and permeabilised for 10 min with 0.1% Triton X-100/PBS. Cells were subsequently blocked overnight with 3% BSA/PBS. For MTOC imaging, cells were stained with 1 µg/ml anti-pericentrin antibody (ab4448, Abcam) for 2 h at 4°C, followed by 5 µg/ml AF568 labelled anti-rabbit IgG H&L secondary antibody (A11035, Invitrogen). To image F-actin at the synapse, fixed conjugates were stained with 33 nM phalloidin-AF647 or phalloidin-AF488 (A22287 and A12379, Thermo Fisher Scientific) for 1 h at room temperature. To image granule polarisation, cells were stained with anti-LAMP-1-AF647 (5 μg/ml) for 1 h (sc-20011, Santa Cruz Biotechnology). Localisation of talin was determined using anti-talin 1 (MAB1676, Sigma-Aldrich) at 5 μg/ml for 1 h, followed by 5 µg/ml AF568 anti-mouse IgG H&L secondary antibody (A11031, Thermo Fisher Scientific).

Conjugates were imaged by confocal microscopy (Leica TCS SP8) using a 100×/1.40 NA oil-immersion objective and white light laser source. Images were acquired using sequential imaging to avoid spectral overlap and analysed using ImageJ (Schneider et al., 2012; National Institutes of Health). Accumulation of F-actin at the synapse was determined by the fold increase in MFI staining at the cell bead interface divided by the MFI from a region at the back of the cell of the same size. Spreading of NK cells against the beads was assessed by measuring the length of F-actin at the bead/cell interface. Polarisation of the MTOC was assessed by measuring the ratio of the distance from the MTOC to the cell-bead interface to the distance from the synapse to the back of the cell. Granule polarisation was quantified by dividing the section of the cell containing perforin granules by the length of the whole cell. To determine the percentage of polarised NK cells, polarised conjugates were ones in which the MTOC ratio was <0.3 and granules were clustered around the MTOC (scored visually). Conjugates were categorised as kinapses when the distribution of F-actin within the NK cell was asymmetrical, with the greatest accumulation outside the IS. Symmetrical conjugated NK cells with F-actin accumulated at the IS were designated as synapses. Conjugates in which F-actin symmetry was unclear were excluded from this analysis. Images of talin are *z*-projections of 0.3 μm optical slices.

The imaging experiments described throughout this article were restricted to analysis of conjugates between a single cell and a single bead. Any bead that displayed aberrant properties was not scored in these assays; beads that appeared abnormal were entirely excluded. The brightness and contrast of images has been adjusted in representative images to make the MTOC more clearly visible, but all analyses were carried out on unadjusted images.

### Imaging flow cytometry

Beads coated in mAb were washed in PBS and analysed by imaging flow cytometry (ImagestreamX MK-II; Amnis). Both brightfield and fluorescent images were captured using 40× zoom, and data were analysed using the IDEAS software package (Merck). Bead debris and doublets were gated out before bead analysis and a minimum of 5000 beads were acquired per experiment.

### Statistical analysis

All statistical analyses were carried out using GraphPad Prism (GraphPad software, version 7). For each data set, the Shapiro–Wilk normality test was used to evaluate the distribution of values. For groups of data displaying normal Gaussian distributions, the statistical significance was determined by the use of parametric tests. Two-tailed *t*-tests were used to examine statistical significance between two groups of data and one-way ANOVA was used for three or more data groups. To compare statistically significant differences between groups, one-way ANOVA with Tukey's multiple comparisons was carried out. For data not showing normal distribution, non-parametric equivalent tests were used. Wilcoxon signed-rank tests were used as the non-parametric *t*-test equivalent, and the Kruskal–Wallis test or matched values Friedman test with Dunn's post-testing was used to replace the one-way ANOVA. A calculated *P*<0.05 was considered statistically significant (indicated by a single asterisk). *P*<0.01 (**), *P*<0.001 (***) and *P*<0.0001 (****), and *P*≥0.05 was considered not significant (ns).

## Supplementary Material

Supplementary information
