# Supplementary material for: Natural killer cell immune synapse formation and cytotoxicity are controlled by tension of the target interface
Source: J Cell Sci. 2021 Apr 15;134(7):jcs258570. doi: 10.1242/jcs.258570 (PMC8077183; doi:10.1242/jcs.258570)
Supplement: Supplementary information [file joces-134-258570-s1.pdf]

Figure S1.

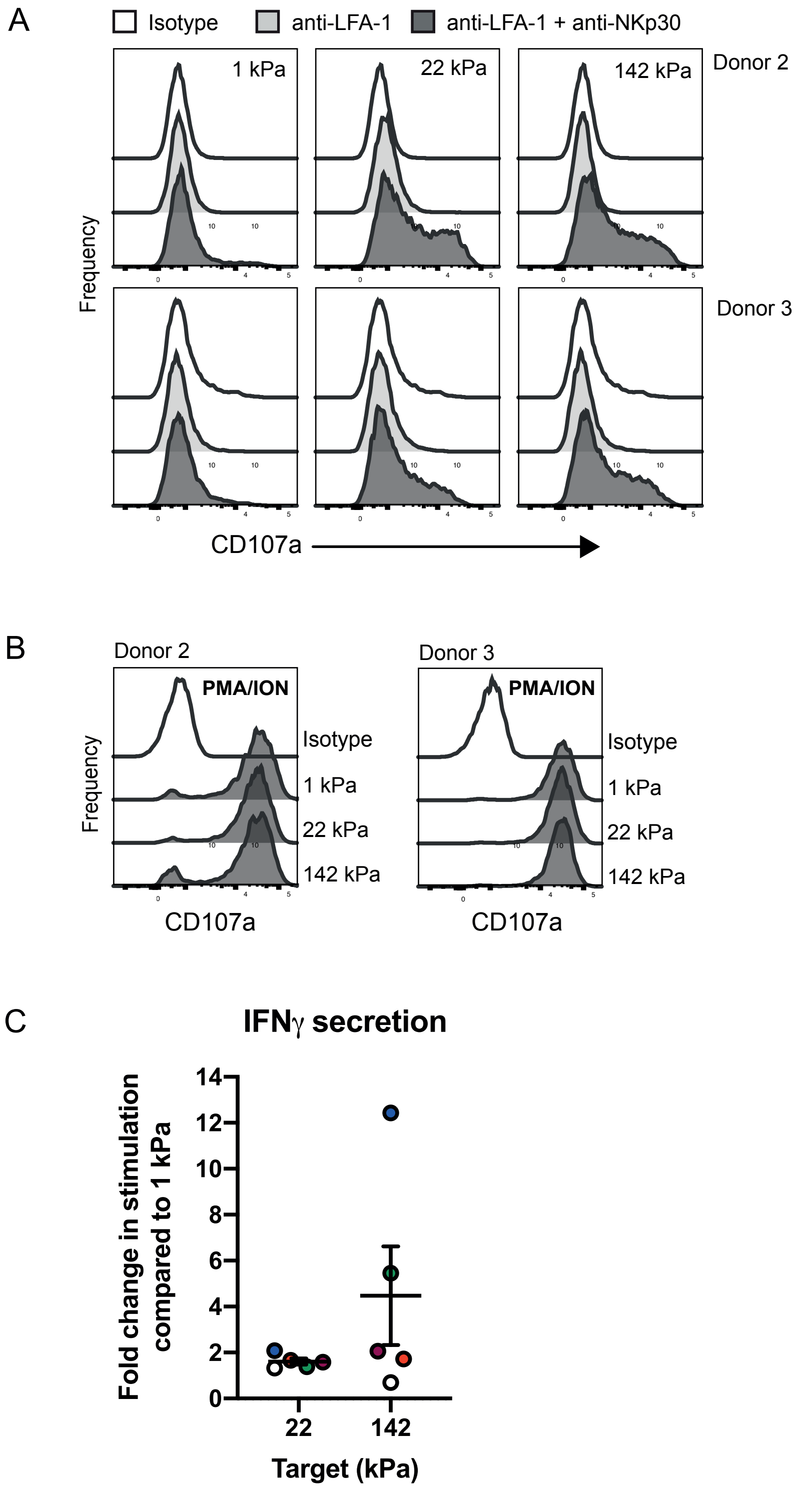

Journal of Cell Science • Supplementary information

Figure S1. NK cell degranulation is sensitive to changes in target stiffness across multipl e donors.

A. Gels coated with anti-LFA-1 mAb alone or in combination with anti-NKp30 mAb were seeded with NK cells and incubated at 37°C for 4 h. Cells were stained for surface expression of CD107a and analysed by flow cytometry. Histograms are shown from two independent donors, in addition to the donor represented in Fig. 1 C. **B.** Primary NK cells were seeded on anti-LFA-1 mAb coated hydrogels for 3 h at 37 °C, treated with both PMA and ionomycin and left for a further 1 h. Cells were stained for surface expression of CD107a and analysed by flow cytometry. Histograms are shown here from two independent donors in addition to the donor represented in Fig. 1. E. **C.** Fold change in each donor's IFNγ response against the 22 and 142 kPa gels in comparison to that donor's response to 1 kPa stiffness gels.

Journal of Cell Science • Supplementary information

Figure S2.

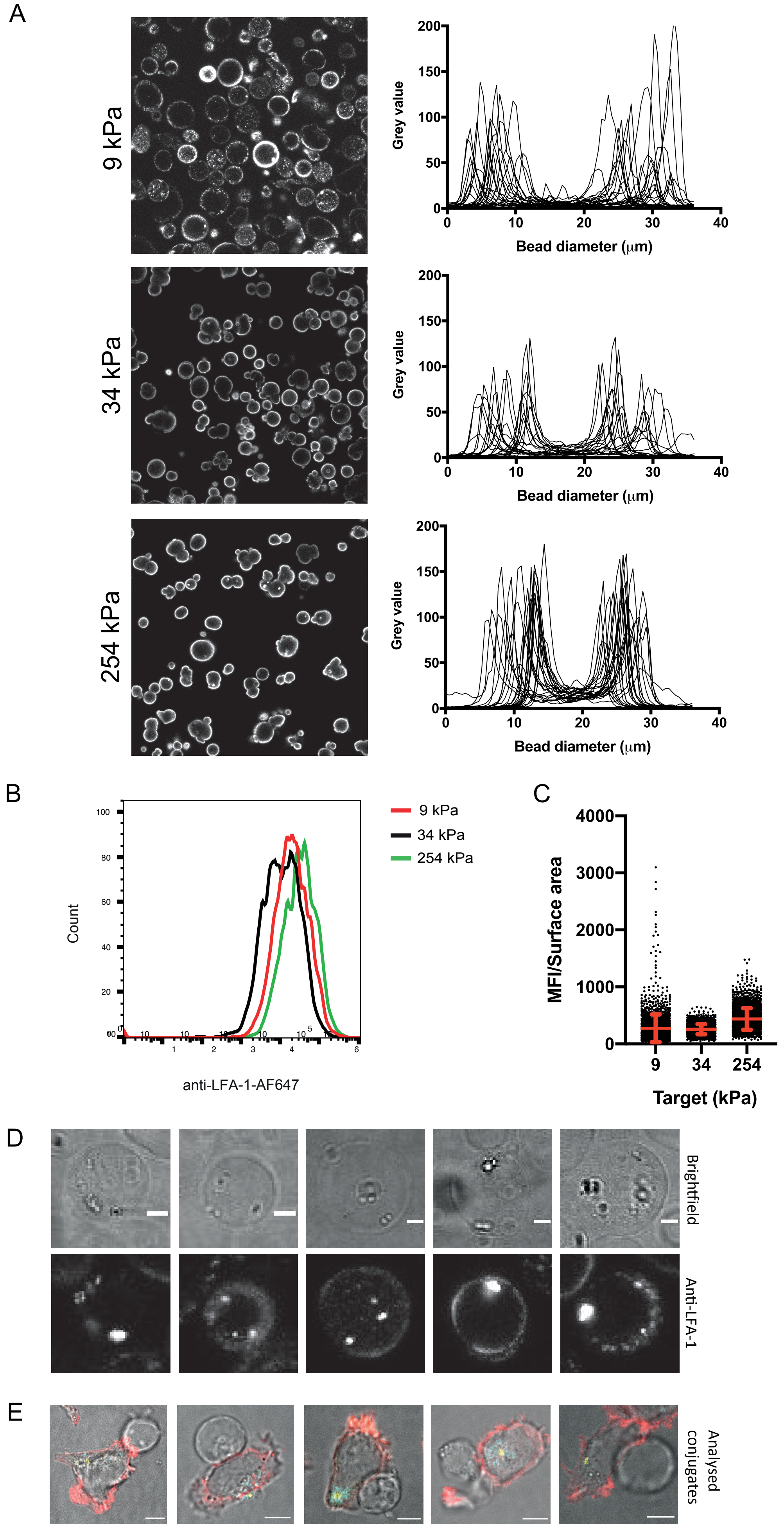

Journal of Cell Science • Supplementary information

Figure S2. Antibody predominantly binds alginate bead surface.

A. Protein binding was characterised in beads coated with anti-NKp30-AF647. Representative confocal images are shown, along with corresponding fluorescence line profile plots. These were drawn by measuring mean fluorescence across the bead diameter and plotting the corresponding intensities (25-30 beads). **B.** 9, 34 and 254 kPa targets were coated with 50, 10 and 10μg/mL anti-LFA-1-AF647 mAb respectively and visualised using imaging flow cytometry. Presented here are representative histogram plots of gMFI values**. C.** The coating density for different bead types was calculated using imaging flow cytometry, relating the diameter of each individual bead to the mean fluorescence intensity of anti-LFA-1. **D.** Representative images showing beads with significant internal staining (brightfield and anti-LFA-1-AF647), which were excluded from analyses (scale = 5μm). **E.** Examples of 9 kPa bead conjugates which were included in the analysis, stained with anti-pericentrin (shown in yellow), phalloidin (shown in red) and anti-CD107a (shown in cyan) (scale = 5μm).

Figure S3.

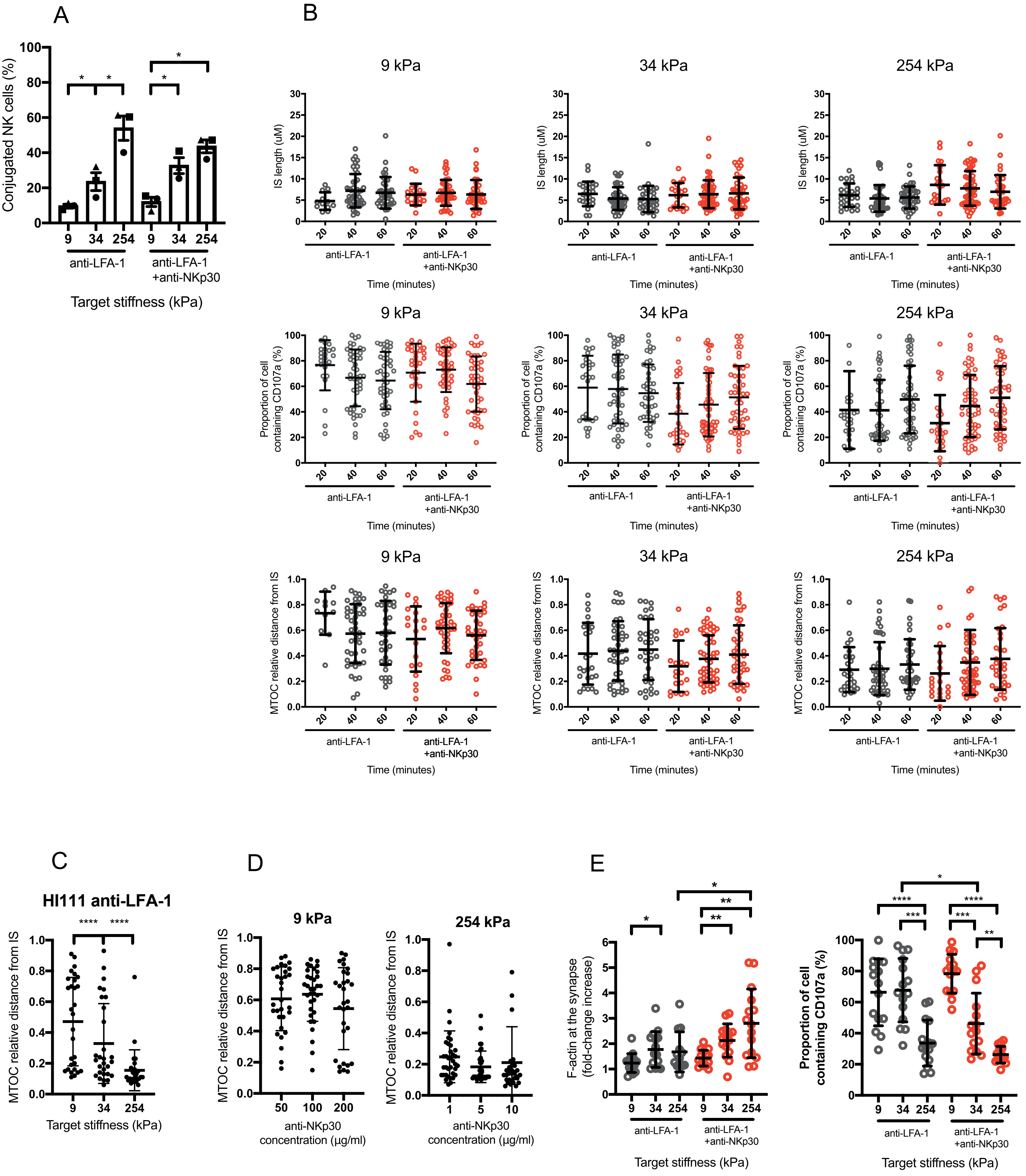

Journal of Cell Science • Supplementary information

Figure S3. Defective NK cell polarisation against soft targets is robust across various controls for mAb specificity, concentration and methodology.

A. 9, 34 and 254 kPa beads were mixed with primary NK cells at a ratio of 1:1 and left to form conjugates for 20 min prior to fixation. Conjugates were imaged using confocal microscopy. Five random locations were selected and proportion of NK cells in conjugates was determined by dividing number of NK cells in contact with a bead by overall number. Data points represent % conjugated NK cells in individual donors (60-180 cells per donor), with error bars showing mean±SEM. P<0.05 *. **B.** Targets were mixed with primary NK cells and left to form conjugates for 20, 40, and 60 min prior to fixation. The IS length was quantified by overlaying the fluorescence image with the corresponding brightfield image and measuring the synapse. The proportion of the cell containing granules was quantified by dividing the length of the cell containing granules by the total length of the cell. The distance of the interface to the MTOC divided by the length of the NK cell gave a ratio value to quantify polarisation of the MTOC. Lower values denote greater polarisation. Data points on graphs represent individual conjugates (3-10 per donor) pooled from independent donors (n=3), with error bars showing mean±SD. **C.** Primary NK cells were mixed with targets coated with an alternative anti-LFA-1 mAb clone, HI111, in combination with anti-NKp30 mAb and incubated at 37°C for 20 min. The fixed conjugates were stained and imaged using confocal microscopy. The ratio value to quantify MTOC polarisation was calculated by dividing the distance of the interface to the MTOC by the length of the NK cell. Data points on graphs represent individual conjugates (9-11 per donor) pooled from independent donors (n=3), with error bars showing mean±SD. **** P<0.0001. **D.** Anti-NKp30 mAb was added to 9 kPa beads at a concentration of 50, 100 and 200 µg/mL, and anti-LFA-1 mAb at 50 µg/mL. To 254 kPa beads, anti-NKp30 mAb was added at 1, 5 and 10 µg/mL with 10 µg/mL anti-LFA-1 mAb. These targets were mixed with primary NK cells at a 1:1 ratio, and MTOC polarisation was quantified. Data points on graphs represent individual conjugates (9-16 per donor) pooled from independent donors (n=3), with error bars showing mean±SD. **E.** 9, 34 and 254 kPa targets were mixed with primary NK cells at a ratio of 1:1 and left to form conjugates for 20 min. Fixed conjugates were

Journal of Cell Science • Supplementary information

stained with phalloidin-AF488 and anti-LAMP-1-AF647. The conjugates were imaged in 3D, with 0.3 µm z-slices spanning the entire NK cell. An image was created representing the sum of all fluorescence in each channel. The amount of f-actin present was quantified by measuring the mean fluorescence intensity of f-actin at the synapse and dividing it by the mean fluorescence intensity of an equivalent sized region of the membrane at the back of the cell. The proportion of the cell containing granules was quantified by dividing the distance between the IS and furthest granule by the total length of the cell. Each data point represents one conjugate, with 5 conjugates per donor (n=3) and error bars showing mean±SD. * $P<0.05$, ** $P<0.01$, *** $P<0.001$, **** $P<0.0001$.

Journal of Cell Science • Supplementary information

Figure S4.

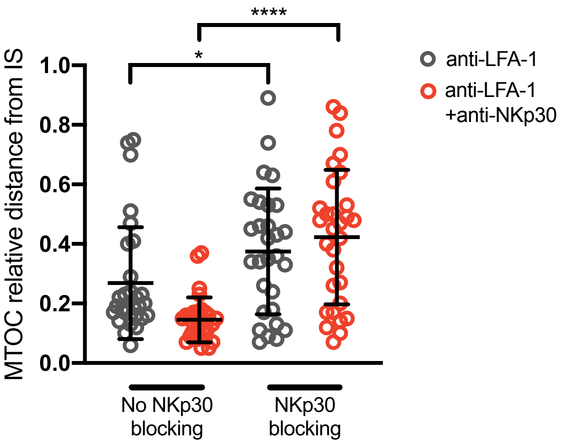

Figure S4. Blocking NKp30 abrogates MTOC polarisation against 254 kPa targets.

To block the NKp30 receptor, primary NK cells were pre-treated with either an IgG1 isotype-matched control mAb or anti-NKp30 mAb for 1 h at 37 ˚C. NK cells were subsequently mixed with beads coated with anti-LFA-1 mAb or anti-LFA-1 and anti-NKp30 mAb at a 1:1 ratio. Cells were fixed and stained, and MTOC polarisation was quantified as before. Data points on graphs represent individual conjugates (9-11 per donor) pooled from 3 independent donors with error bars showing mean±SD. * $P<0.05$, **** $P<0.0001$.

Journal of Cell Science • Supplementary information

Figure S5.

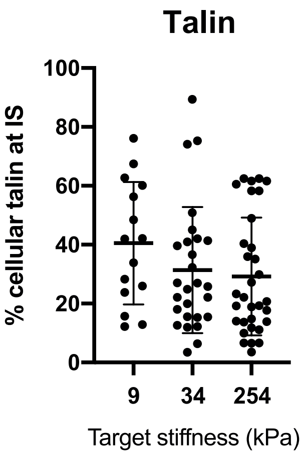

Figure S5. Accumulation of cellular talin at synapses is similar across all target stiffnesses.

Primary NK cells were mixed at a 1:1 ratio with 9, 34 and 254 kPa beads coated in anti-LFA-1 and anti-NKp30 mAbs and left to form conjugates for 20 min. After fixing, conjugates were stained with anti-talin mAb with goat anti-mouse IgG (H+L) AF568 secondary antibody and phalloidin-AF647, and then imaged using confocal microscopy. The conjugates were imaged in 3D, with 0.3 µm z-slices spanning the entire NK cell. An image was created representing the sum of all talin fluorescence. The percentage of cellular talin present at the contact point was quantified by measuring the mean fluorescence intensity of talin in the contact region and dividing it by the mean fluorescence intensity of talin in the whole cell. Data points on graphs represent individual conjugates (4-10 per donor) pooled from 3 independent donors with error bars showing mean±SD.

Journal of Cell Science • Supplementary information

Figure S6.

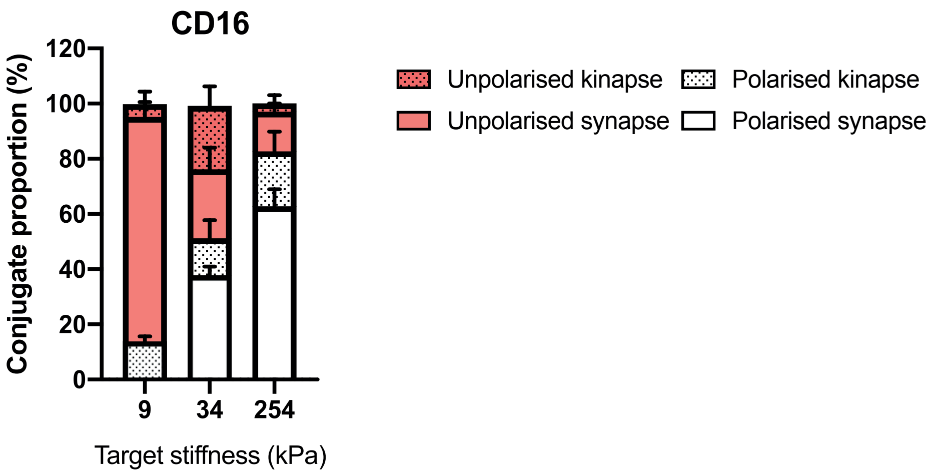

Figure S6. Target stiffness determines stability of synapse through CD16.

9, 34 and 254 kPa targets were coated with 50, 10 and 10μg/mL anti-CD16 3G8 mAb and anti-LFA-1, respectively. Targets were mixed with NK cells at a 1:1 ratio and incubated for 20 min at 37°C. Conjugates were stained, imaged using confocal microscopy then scored on MTOC polarisation and localisation of f-actin. MTOC relative distance from IS values <0.3 were denoted as polarised. NK cells were categorised as forming a kinapse when f-actin was asymmetrically distributed relative to the point of contact. All conjugates in which the degree of symmetry was unclear were discarded (<5%). Bars represent mean of three donors (8-12 conjugates per donor) with error bars showing mean±SD.

Journal of Cell Science • Supplementary information
